# Plasma Heat Shock Protein 70 Is Associated With the Onset of Acute Myocardial Infarction and Total Occlusion in Target Vessels

**DOI:** 10.3389/fcvm.2021.688702

**Published:** 2021-09-23

**Authors:** Runda Wu, Wei Gao, Zheng Dong, Ya Su, Yuyao Ji, Jianquan Liao, Yuanji Ma, Yuxiang Dai, Kang Yao, Junbo Ge

**Affiliations:** ^1^Department of Cardiology, Zhongshan Hospital, Fudan University, Shanghai, China; ^2^Shanghai Institute of Cardiovascular Disease, Shanghai, China; ^3^National Health Commission Key Laboratory of Viral Heart Diseases, Fudan University, Shanghai, China; ^4^Key Laboratory of Viral Heart Diseases, Chinese Academy of Medical Sciences, Beijing, China

**Keywords:** acute myocardial infarction, heat shock protein 70, chronic total occlusion, ST-elevation myocardial infarction, cardioprotection

## Abstract

**Background:** Whether the role of plasma heat shock protein 70 (HSP70) in acute myocardial infarction (AMI) is protective or detrimental remains debated, and the relationship between HSP70 and total occlusion remains elusive.

**Methods:** A total of 112 patients with primary diagnosis of AMI and 52 patients with chronic coronary syndrome (CCS) were enrolled into the study. Plasma HSP70 level was determined by ELISA on day 1 and day 7 after the onset of AMI and was examined before angiography in patients with CCS. Peak NT-proBNP, high-sensitivity C-reactive protein (CRP), troponin T (cTnT), and left ventricular ejection fraction were measured.

**Results:** Plasma HSP70 was significantly higher in CCS than AMI (*P* < 0.0001), and it showed a significant decrease from day 1 to day 7 after AMI (*P* < 0.01). Elevated HSP70 was associated with decreased levels of LDL-C (*P* < 0.05), peak cTnT (*R* = −0.3578, *P* < 0.0001), peak NT-proBNP (*R* = −0.3583, *P* < 0.0001), and peak CRP (*R* = −0.3539, *P* < 0.0001) and a lower diagnosis of AMI (*R* = −0.4016, *P* < 0.0001) and STEMI (*R* = −0.3675, *P* < 0.0001), but a higher diagnosis of total occlusion in target vessels (*R* = 0.1702, *P* < 0.05). HSP70 may provide certain predictive value for the diagnosis of AMI, STEMI, and total occlusion in target vessels, and the area under the receiver operating characteristic curves were 0.7660, 0.7152, and 0.5984, respectively. HSP70 was also negatively associated with in-hospital stay (*P* < 0.001) and positively correlated with left ventricular ejection fraction (LVEF) at 1-year follow-up (*P* < 0.05), despite no association with in-hospital major adverse cardiovascular events (MACE).

**Conclusion:** Plasma HSP70 level was found to decrease from day 1 to day 7 post-AMI, but the overall level of patients with AMI was lower than that of patients with CCS. However, the ability of HSP70 to identify clinically significant AMI and STEMI was moderate, and the predictive value to total occlusion was slight.

## Introduction

Heat shock proteins (HSPs) are known as molecular chaperones that function in protein folding (1), usually induced by hyperthermia, which may also indicate other forms of cellular stress such as lipopolysaccharide, high blood pressure, infection (2), and ischemic injury (1). Despite the view that HSPs may act as damage-associated molecular patterns (DAMPs) during the ischemic damage to interact with pattern recognition receptors (PRRs) and induce a strong immune response in acute myocardial infarction (AMI), HSPs are not bona fide intracellular molecules that normally are not exposed to an extracellular environment (3), and they are detectable in normal circulation. Heat shock protein 70 (HSP70) is one of the members of HSPs, which both regulates intracellular homeostasis and can be released to the extracellular milieu in response to AMI (4, 5).

From the perspective of intracellular housekeeper, increased HSP70 is beneficial to attenuate injury and improve recovery after AMI. Repression of HSP70 may abrogate the protection against ischemic injury in adult cardiomyocytes (6). *In vivo* studies found that overexpression of HSP72, which also refers to HSP70, could improve functional recovery of hearts and decrease infarct size after myocardial ischemia and reperfusion (IR) (7). Exercise-induced HSP70 markedly improved post-ischemic left ventricular function, albeit in male rats (8). Moreover, a single intravenous dose of HSP72 fusion protein could also contribute to strongly reduced myocardial apoptosis and improved cardiac function after IR in rabbits (9). Other studies using HSP co-inducers to increase HSP70 as well led to decreased infarct size during IR in AMI (10–12).

On the contrary, studies focusing on the circulating levels of HSP70 have implicated that it may confer risk for the healing of AMI. Elevated circulating HSP70 was often suggested as a marker of myocardial damage after AMI, which was associated with inflammatory responses (13), and may also promote the progression of heart failure (14). Increased serum levels of HSP72, which is considered as the most important member of the HSP70 family of proteins, and inflammatory markers may also correlate with the degree of cardiac and microvascular dysfunction in patients with angiographically normal coronary arteries (10). Furthermore, HSP70 was significantly increased after AMI and was directly associated with adverse cardiac events and all-cause mortality in the long term (5). Nevertheless, some population studies showed that high levels of HSP70 may have atheroprotective effects (4, 15), and exosomes isolated from healthy humans may exert cardioprotection in cardiac IR via HSP70-mediated effects (16).

The role of HSP70 in AMI is therefore complex and ambiguous (3); whether circulating HSP70 could be utilized as a biomarker and whether the marker is friend or foe after AMI remains debated. The association of HSP70 with features of lesions in coronary artery disease (CAD) remains uncertain. In this study, we determined the release of HSP70 of patients on day 1 and day 7 post-AMI. We also analyzed its association with the onset of AMI, ST segment-elevation myocardial infarction (STEMI), features of lesions, and biomarkers that reflect severity of myocardial infarction (MI).

## Methods

### Study Patients

We performed an observational, single-center cohort study. A total of 175 patients with complaints of “chest pain and discomfort” were referred to Zhongshan Hospital, Fudan University, and finally, 164 patients were included after the exclusion of autoimmune diseases, infections, and malignancy. Among the patients consecutively enrolled, 112 patients were diagnosed with AMI by the fourth universal definition of myocardial infarction (17), and 52 patients were classified into chronic coronary syndrome (CCS) group accordingly (18). In detail, the patients in the CCS group referred to those with stable angina and >70% coronary stenosis on at least one main coronary artery by angiography. All patients underwent coronary angiography after admission and fasting venous blood was drawn during hospitalization. The AMI group was further divided into two subgroups: day 1 post-MI (MI1d, *n* = 60) when the ischemic injury begins expanding and day 7 post-MI (MI7d, *n* = 92) when the extent of injury is presumed to gradually decline and wound healing is started, with 40 patients in both subgroups who were self-paired. The CCS group was classified by angiography into patients with no chronic total occlusion (CCS-NCTO, *n* = 24) and with chronic total occlusion (CCS-CTO, *n* = 28) in target vessels. Informed consent was obtained from all patients and the study was approved by the ethical committee.

### Patient and Public Involvement

Patients or the public were not involved in the design, or conduct, or reporting, or dissemination plans of our research.

### Assessment of Coronary Lesions

During the coronary angiography after enrollment within 24–72 h upon admission, the procedure was performed by an experienced cardiologist and the angiograms were reviewed by two separate cardiologists blinded with the level of circulating HSP70. Revascularization was performed according to the guideline (17, 18). The assessment of coronary lesions included the assumed culprit or most diseased vessels, the stenosis of culprit or most diseased vessels, whether there was a total occlusion in culprit vessels, whether thrombosis of culprit vessels occurred, and the number of diseased vessels.

### Measurement of Plasma HSP70

Whole-blood samples with anticoagulation of ethylene diamine tetraacetic acid were drawn on day 1 and day 7 post-MI, and in patients with CCS, the blood was drawn before angiography. The plasma was isolated by centrifugation at 4°C, 3,000 rpm for 15 min. The plasma HSP70 was tested by immunoassay (HSP70 high sensitivity ELISA kit, ADI-EKS-715, Enzo Life, USA) within 6 h upon isolation. The measuring range is 0.2–12.5 ng/ml, and the coefficient of variation of the assay ranged 2.8–11.3%.

### Study Outcomes

The clinical primary outcome of the study was in-hospital major adverse cardiovascular events (MACE), which included cardiac death, non-fatal AMI, repeat revascularization, stroke, malignant ventricular arrhythmia including ventricular tachycardia and fibrillation, and acute left ventricular failure. Other endpoints included in-hospital stay and left ventricular ejection fraction (LVEF) both in-hospital and at 1-year follow-up.

### Statistical Analysis

Abnormally distributed data were expressed as median and normally distributed data were expressed as mean ± SD. Difference of quantitative data between groups was compared by one-way ANOVA and Student's *t*-test, and enumeration data were compared by chi-square test. Kruskal–Wallis test, Mann–Whitney *U* test, and Wilcoxon matched-pairs signed rank test of paired data were used to detect the difference between groups considering the level of plasma HSP70 was not normally distributed. Spearman correlation, simple linear regression analysis, and simple logistic regression were performed to assess the relationships between plasma HSP70 and peak value of N-terminal pro-B-type natriuretic peptide (NT-proBNP), peak C-reactive protein (CRP), and peak cardiac troponin T (cTnT) and the diagnosis of AMI, STEMI, and the total occlusion of target vessels. Specifically, simple logistic regression analysis was conducted for HSP70 as potential predictors for MACE, and the correlation of HSP70 and other outcomes was conducted by Spearman correlation analysis. As to the evaluation of diagnostic ability of HSP70 to STEMI and total occlusion, receiver operating characteristic (ROC) curve analysis was performed.

## Results

### Baseline Data

Among the 164 patients, 138 patients were found to have luminal stenosis over 75% in at least one main coronary artery by angiography, with 127 patients receiving percutaneous coronary intervention (PCI) and 11 receiving coronary artery bypass graft (CABG), and the demographic characteristics and assessment of coronary lesions are displayed in Tables 1–3, respectively. It is demonstrated that the plasma levels of HSP70, age, history of previous MI, administration of statins, total cholesterol (TC), low-density lipoprotein-cholesterol (LDL-C), high-density lipoprotein-cholesterol (HDL-C), D-dimer, peak cTnT level, peak NT-proBNP, peak CRP, and LVEF were different among the four subgroups. Considering the pathologic disparity between acute occlusion and chronic occlusion, a detailed comparison was conducted among the three subgroups, MI1d, MI7d, and CCS-NCTO, and it was found that only plasma HSP70 (*P* = 0.0015), statin use (*P* = 0.0342), TC (*P* = 0.0013), LDL-C (*P* = 0.0004), D-dimer (*P* < 0.0001), p-cTnT (*P* < 0.0001), p-NT-proBNP (*P* < 0.0001), p-CRP (*P* < 0.0001), and LVEF (*P* < 0.0001) were significantly different. As to the conditions of coronary lesions, it was shown that the diagnoses of total occlusion in target vessel (*P* = 0.0003) and target vessel stenosis (*P* < 0.0001) were significantly different among the aforementioned three groups.

**Table 1:** Population characteristics of the MI and CCS groups.

	**MI (*n* = 112)**	**CCS (*n* = 52)**	***P*-value**
Age (years)	65.8 ± 11.9	59.7 ± 14.2	<0.01
Male sex	95 (84.8)	45 (86.5)	0.7736
BMI	26.80 ± 11.31	24.83 ± 2.60	0.4043
Diabetes	31 (27.7)	19 (36.5)	0.2541
Hypertension	72 (64.3)	33 (63.5)	0.9191
Stroke	8 (7.1)	1 (1.9)	0.1741
Previous MI	11 (9.8)	14 (26.9)	<0.01
Family history of CAD	13 (11.6)	2 (3.8)	0.1099
Smokers	56 (50.0)	19 (36.5)	0.1086
Drinkers	10 (8.9)	2 (3.8)	0.2475
Statins	51 (45.5)	39 (76.5)	<0.001
Nitrates	21 (18.8)	8 (15.4)	0.6018
STEMI diagnosis	68 (60.7)	–	–
TC (mmol/L)	4.48 ± 1.15	3.62 ± 0.82	<0.0001
TG (mmol/L)	1.96 ± 1.03	1.90 ± 0.92	0.7540
LDL-C (mmol/L)	2.54 ± 1.11	1.77 ± 0.77	<0.001
HDL-C (mmol/L)	1.01 ± 0.26	1.02 ± 0.30	0.9543
D-dimer (mg/L)^a^	0.86 (0.29, 1.29)	0.20 (0.19, 0.30)	<0.01
p-cTnT (ng/ml)^a^	3.24 (1.52, 7.28)	0.01 (0.01, 0.02)	<0.0001
p-NT-proBNP (pg/ml)^a^	1,471 (871.8, 4,480)	124.5 (54.2, 366.2)	<0.0001
p-CRP (mg/L)^a^	33.55 (10.45, 81.15)	0.45 (0.15, 2.15)	<0.0001
Plasma HSP70 (ng/ml)^a^	0.42 (0.26, 0.80)	1.02 (0.55, 1.63)	<0.001
LVEF (%)	50 ± 10	62 ± 8	<0.0001

*Values are displayed as mean ± SD or n (%), unless otherwise indicated. The level of plasma HSP70 in paired patients with MI was calculated as the level in 1 day post-MI*.

*BMI, body mass index; MI, myocardial infarction; CAD, coronary artery disease; STEMI, ST-segment elevation myocardial infarction; TC, total cholesterol; TG, triglyceride; LDL-C, low-density lipoprotein-cholesterol; HDL-C, high-density lipoprotein-cholesterol; p-cTnT, peak level of cardiac troponin T; p-NT-proBNP, peak level of N-terminal pro-B-type natriuretic peptide; p-CRP, peak level of C-reactive protein; HSP70, heat shock protein 70; LVEF, left ventricular ejection fraction*.

a *Median and interquartile range*.

**Table 2:** Population characteristics of the four subgroups.

	**MI1d (*n* = 60)**	**MI7d (*n* = 92)**	**CCS-NCTO (*n* = 24)**	**CCS-CTO (*n* = 28)**	***P*-value**
Age (years)	64.6 ± 10.9	66.1 ± 11.9	65.9 ± 13.5	54.4 ± 12.6	0.0001
Male sex	51 (85)	80 (87)	18 (75)	27 (96.4)	0.1623
BMI	23.81 ± 2.77	23.88 ± 3.16	24.41 ± 2.93	25.43 ± 2.07	0.4293
Diabetes	16 (26.7)	22 (23.9)	10 (41.7)	9 (32.1)	0.3503
Hypertension	35 (58.3)	60 (65.2)	18 (75)	15 (53.6)	0.3490
Stroke	4 (6.7)	7 (7.6)	1 (4.2)	0 (0)	0.4863
Previous MI	6 (10)	9 (9.8)	4 (16.7)	10 (35.7)	0.0045
Family history of CAD	9 (15)	12 (13)	1 (4.2)	1 (3.6)	0.2622
Smokers	32 (53.3)	47 (51.1)	7 (29.2)	12 (42.9)	0.1917
Drinkers	5 (8.3)	9 (9.8)	0 (0)	2 (7.1)	0.4645
Statins	33 (55)	42 (45.7)	18 (75)	21 (75)	0.0088
Nitrates	13 (21.7)	16 (17.4)	7 (29.2)	1 (3.6)	0.0895
STEMI diagnosis	37 (61.7)	60 (65.2)	–	–	0.6561
TC (mmol/L)	4.72 ± 1.10	4.39 ± 1.08	3.62 ± 0.89	3.61 ± 0.75	0.0001
TG (mmol/L)	2.16 ± 1.13	1.79 ± 0.86	2.02 ± 1.09	1.78 ± 0.70	0.4532
LDL-C (mmol/L)	2.73 ± 1.19	2.48 ± 0.98	1.64 ± 0.79	1.93 ± 0.73	0.0003
HDL-C (mmol/L)	0.97 ± 0.24	1.03 ± 0.25	1.14 ± 0.34	0.90 ± 0.21	0.0120
D-dimer (mg/L)^a^	0.82 (0.28, 1.12)	0.93 (0.37, 1.45)	0.20 (0.19, 0.29)	0.19 (0.19, 0.32)	<0.0001
p-cTnT (ng/ml)^a^	2.85 (1.16, 5.78)	3.95 (2.01, 8.14)	0.009 (0.005, 0.013)	0.015 (0.008, 0.052)	<0.0001
p-NT-proBNP (pg/ml)^a^	1,054 (687, 3,195)	1,598 (892, 5,289)	73 (40, 237)	155 (72, 446)	<0.0001
p-CRP (mg/L)^a^	20.6 (8.6, 43.6)	41.5 (14.6, 89.8)	0.2 (0.2, 0.5)	1.8 (0.7, 4.9)	<0.0001
Plasma HSP70 (ng/ml)^a^	0.54 (0.27, 0.86)	0.41 (0.25, 0.69)	0.85 (0.48, 1.39)	1.10 (0.75, 1.83)	<0.0001
LVEF (%)	51 ± 10	49 ± 10	66 ± 4	58 ± 9	<0.0001

*Values are displayed as mean ± SD or n (%), unless otherwise indicated*.

*BMI, body mass index; MI, myocardial infarction; CAD, coronary artery disease; STEMI, ST-segment elevation myocardial infarction; TC, total cholesterol; TG, triglyceride; LDL-C, low-density lipoprotein-cholesterol; HDL-C, high-density lipoprotein-cholesterol; p-cTnT, peak level of cardiac troponin T; p-NT-proBNP, peak level of N-terminal pro-B-type natriuretic peptide; p-CRP, peak level of C-reactive protein; HSP70, heat shock protein 70; LVEF, left ventricular ejection fraction*.

a *Median and interquartile range*.

**Table 3:** The assessment of coronary lesions in the four groups.

	**MI1d (*n* = 60)**	**MI7d (*n* = 92)**	**CCS-NCTO (*n* = 24)**	**CCS-CTO (*n* = 28)**	***P*-value**
Culprit vessel or most diseased vessel is LAD	26 (43.3)	45 (48.9)	17 (70.8)	12 (42.9)	0.0697
Culprit vessel or most diseased vessel is RCA	17 (28.3)	25 (27.2)	6 (25)	13 (46.4)	
Culprit vessel or most diseased vessel is LCX	12 (20)	11 (12)	1 (4.2)	3 (10.7)	
Total occlusion in target vessel	25 (41.7)	40 (43.5)	–	28 (100)	–
Target vessel stenosis (%)^a^	99 (95, 100)	99 (90, 100)	82.5 (62.5, 90)	100 (100, 100)	<0.0001
Thrombosis of target vessel	14 (23.3)	16 (17.4)	–	–	0.3683
Number of diseased vessels^a^	3 (2, 3)	3 (2, 3)	3 (2, 3)	3 (3, 3)	0.0960
PCI	48 (80)	70 (76)	16 (66.7)	26 (92.9)	0.0497
CABG	4 (6.7)	9 (9.8)	0 (0)	1 (3.6)	

*Values are displayed as mean ± SD or n (%), unless otherwise indicated.*

*LAD, left anterior descending branch of coronary artery; RCA, right coronary artery; LCX, left circumflex branch of coronary artery; PCI, percutaneous coronary intervention; CABG, coronary artery bypass graft*.

a *Median and interquartile range*.

### Detailed Comparison of Plasma HSP70

**Figure 1:**
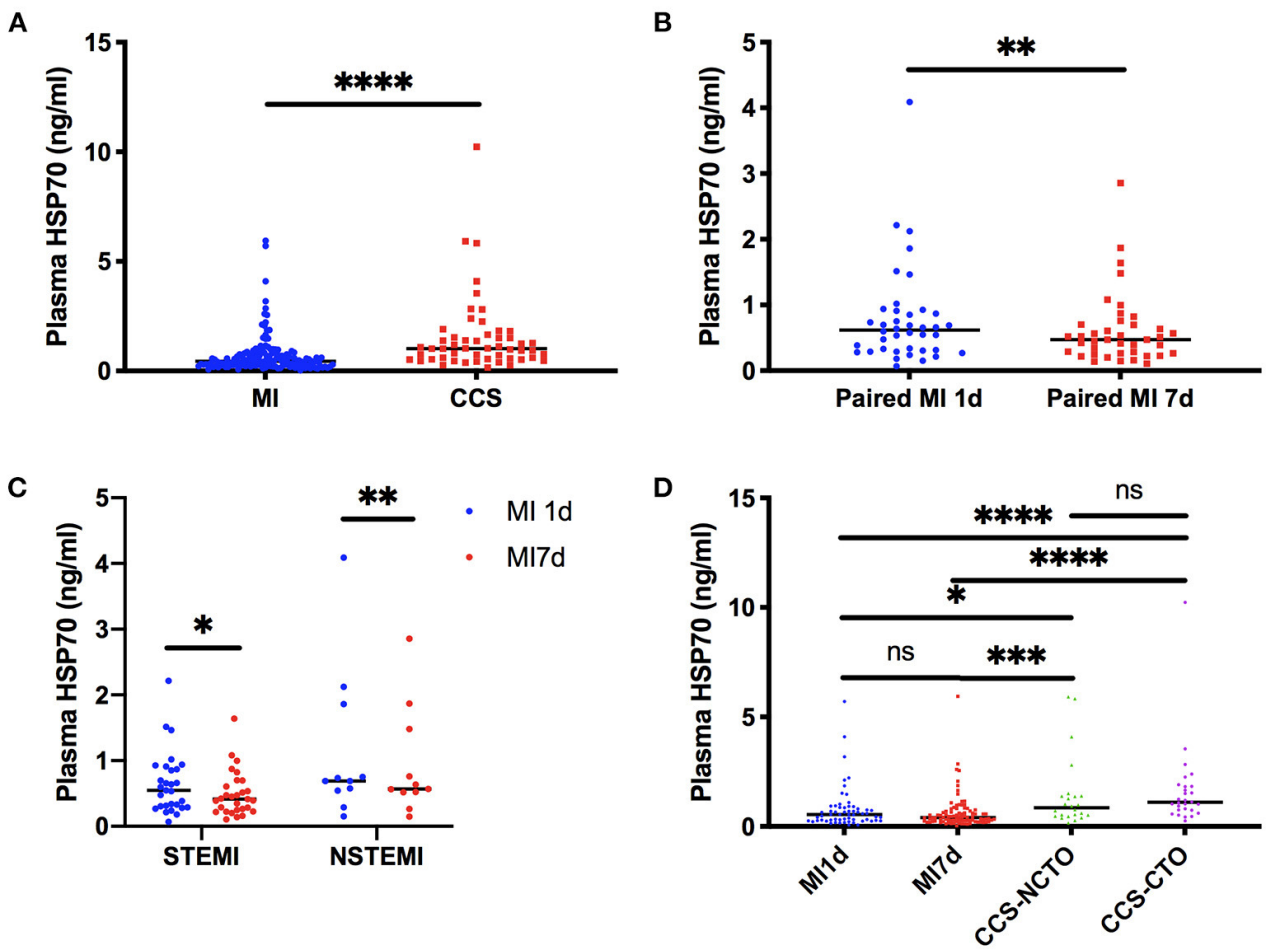
Detailed comparison of plasma HSP70. **(A)** Comparison between overall patients with MI and CCS. **(B)** Matched-pairs comparison between day 1 and day 7 post-MI. **(C)** Matched-pairs comparison between day 1 and day 7 in STEMI and NSTEMI. **(D)** Comparison among patients with day 1 and day 7 post-MI, CCS with no CTO, and CCS with CTO lesions. *P < 0.05, **P < 0.01, ***P < 0.001, ****P < 0.0001. ns, not significant; MI, myocardial infarction; MI1d, 1 day post-MI; MI7d, 7 days post-MI; STEMI, ST-segment elevation myocardial infarction; NSTEMI, non-ST-segment elevation myocardial infarction; CCS, chronic coronary syndrome; CTO, chronic total occlusion; NCTO, no chronic total occlusion.

When compared as total, the HSP70 level of patients with MI [median 0.44, interquartile range (IQR) 0.26–0.76] was significantly lower than CCS (1.02, 0.55–1.63, *P* < 0.0001, Figure 1A and Table 4). Although the difference of HSP70 level between day 1 and day 7 post-MI was not significant (*P* = 0.1708), which may be due to the small sample size, in the paired comparison of 40 patients whose levels of HSP70 were examined both on day 1 and day 7 after MI, it showed a marked decline of HSP70 over time upon the onset of MI [MI1d, 0.62 (0.31–0.90); MI7d, 0.47 (0.26–0.70), *P* = 0.0016, Figure 1B], which is consistent with previous studies. Specifically, there existed no significant difference on day 1 and day 7 post-MI in total patients diagnosed with STEMI (*P* = 0.0627) and NSTEMI (*P* = 0.9225), whereas in paired patients, HSP70 was still lower on day 7 than day 1 in both STEMI [MI1d, 0.55 (0.30–0.89); MI7d, 0.42 (0.24–0.65), *P* = 0.0365] and NSTEMI [MI1d, 0.69 (0.54–1.86); MI7d, 0.57 (0.52–1.48), *P* = 0.0020, Figure 1C] patients. A detailed comparison of HSP70 among the four groups is displayed in Figure 1D, with HSP70 remarkably higher in CCS-NCTO and CCS-CTO groups than in MI subgroups (*P* < 0.05).

**Table 4:** Comparison of plasma HSP70 among different groups corresponding to Figure 1.

	**Plasma HSP70 (ng/ml)**	***P*-value**
Figure 1A: overall MI (*n* = 152)^a^	0.44 (0.26, 0.76)	<0.0001
Figure 1A: overall CCS (*n* = 52)	1.02 (0.55, 1.63)	
Figure 1B: paired MI1d (*n* = 40)	0.62 (0.31, 0.9)	<0.01
Figure 1B: paired MI7d (*n* = 40)	0.47 (0.26, 0.7)	
Figure 1C: paired STEMI-MI1d (*n* = 29)	0.55 (0.3, 0.89)	<0.05
Figure 1C: paired STEMI-MI7d (*n* = 29)	0.42 (0.24, 0.65)	
Figure 1C: paired NSTEMI-MI1d (*n* = 11)	0.69 (0.54, 1.86)	<0.01
Figure 1C: paired NSTEMI-MI7d (*n* = 11)	0.57 (0.52, 1.48)	
Figure 1D: MI1d (*n* = 60)	0.54 (0.27, 0.86)	0.1708 vs. MI7d; <0.05 vs. CCS-NCTO; <0.0001 vs. CCS-CTO
Figure 1D: MI7d (*n* = 92)	0.41 (0.25, 0.69)	<0.001 vs. CCS-NCTO; <0.0001 vs. CCS-CTO
Figure 1D: CCS-NCTO (*n* = 24)	0.85 (0.48, 1.39)	0.1672 vs. CCS-CTO
Figure 1D: CCS-CTO (*n* = 28)	1.10 (0.75, 1.83)	

*Values are displayed as median and interquartile range*.

*MI, myocardial infarction; MI1d, 1 day post-MI; MI7d, 7 days post-MI; STEMI, ST-segment elevation myocardial infarction; NSTEMI, non-ST-segment elevation myocardial infarction; CCS, chronic coronary syndrome; CTO, chronic total occlusion; NCTO, no chronic total occlusion*.

a *The overall MI group referred to both MI1d patients and MI 7d patients, which included 40 paired patients with identical characteristics but analyzed separately in two subgroups*.

### Association of Plasma HSP70 and Diagnosis of AMI

We analyzed the correlation of the level of HSP70 and the diagnosis of AMI or not, and the Spearman correlation *R* was −0.4016 [95% confidence interval (CI) −0.5138 to −0.2759, *P* < 0.0001], which indicated that HSP70 may be associated with the onset of AMI and a more stable condition like CCS. Thus, the downward trend in the variation of circulating HSP70 in patients from the first day of AMI showed that the nadir value of HSP70 may reflect the disease severity, and the following correlation and regression analyses were performed in data of MI7d, CCS-NTCO, and CCS-CTO.

### Association of Plasma HSP70 and Lipid Levels

As mentioned above, TC, LDL-C, and statin use were significantly different among the groups. However, HSP70 was only negatively correlated with LDL-C (Spearman *R* = −0.2543, 95% CI −0.4443 to −0.04234, *P* = 0.0162) and positively correlated with statin use before admission (Spearman *R* = 0.2212, 95% CI 0.05429 to 0.3760, *P* = 0.0079). These findings suggest that HSP70 might be associated with atherosclerosis, as LDL-C is known to accelerate atherosclerosis development and contribute to plaque instability (19), which can be reversed partly by statins (20).

### Association of Plasma HSP70 and Markers Reflecting Severity of MI

**Figure 2:**
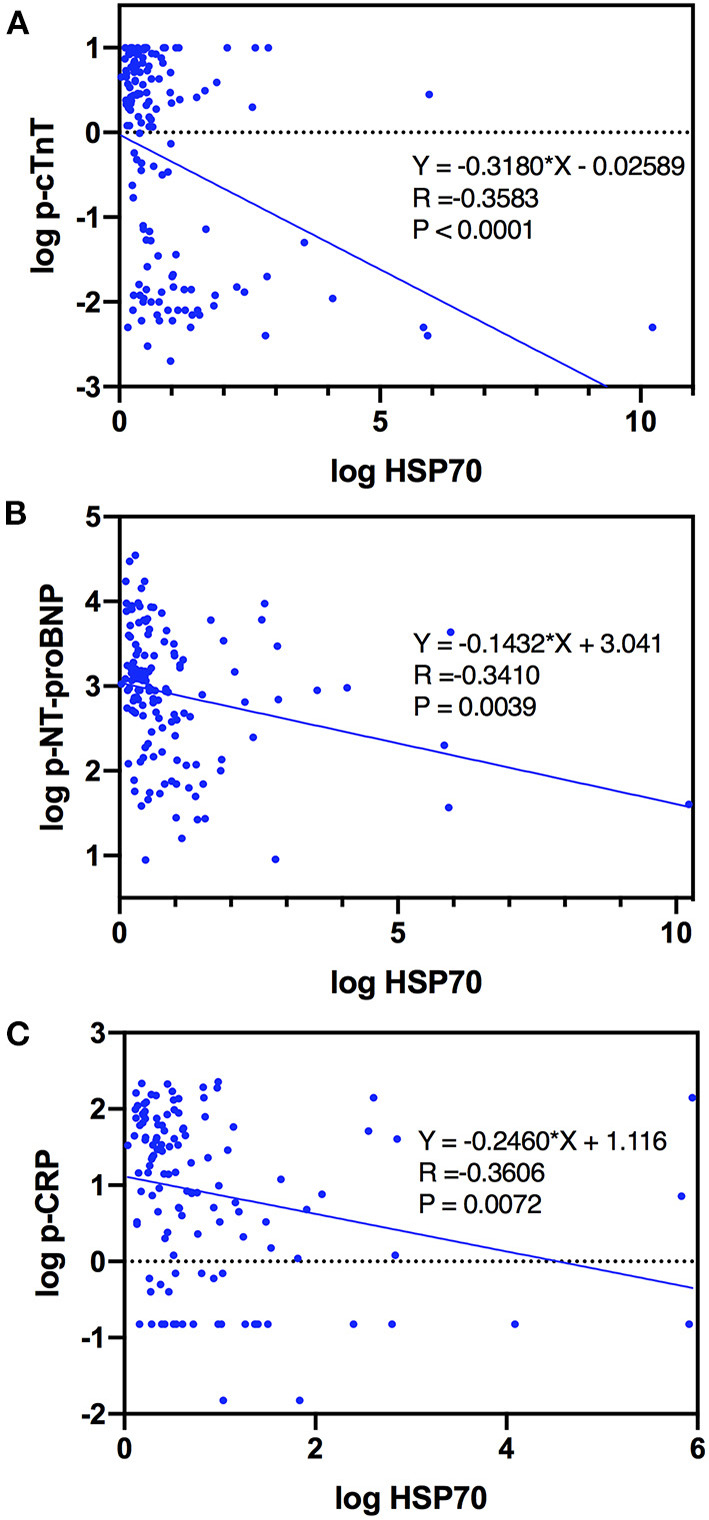
Curve estimations of log HSP70 with **(A)** log p-cTnT, **(B)** log p-NT-proBNP, and **(C)** log p-CRP.

With respect to specific markers as indicators of severity of AMI, which were widely acknowledged, cTnT, NT-proBNP, CRP, and LVEF, we demonstrated that plasma HSP70 was strongly and negatively correlated with p-cTnT (Spearman *R* = −0.3578, 95% CI −0.4976 to −0.1998, *P* < 0.0001), p-NT-proBNP (Spearman *R* = −0.3583, 95% CI −0.4994 to −0.1985, *P* < 0.0001), and p-CRP (Spearman *R* = −0.3539, 95% CI −0.5031 to −0.1844, *P* < 0.0001). Furthermore, scatter plots and simple linear regression estimation curves between log HSP70 and log p-cTnT, log p-NT-proBNP, and log p-CRP were conducted, as shown in Figure 2. Surprisingly, HSP70 was nearly correlated with LVEF (*P* = 0.0540), with the correlation coefficient *R* positive, and the level of D-dimer, a marker which may predict in-hospital adverse outcomes in patients with STEMI (21), was not correlated with HSP70. To sum up, the negative correlations with the markers of cardiac injury, cardiac failure, and inflammation, indicate that HSP70 might be associated with cardiac function recovery, anti-inflammatory responses, and healing after myocardial infarction.

### Association of Plasma HSP70 and Conditions of Coronary Lesions

It has been shown that the diagnosis of total occlusion in target vessels and the degree of stenosis in target vessels vary among the groups. Moreover, it was found that HSP70 level only positively correlated with total occlusion of target vessels (Spearman *R* = 0.1702, 95% CI 0.0019 to 0.3291, *P* = 0.0414) and strongly negatively correlated with the diagnosis of STEMI (Spearman *R* = −0.3675, 95% CI −0.5046 to −0.2123, *P* < 0.0001), which is the common cause of acute occlusion of vessels. This indicates that HSP70 might be more associated with the sustainability of homeostasis during a process of chronic occlusion, rather than conferring risk for acute occlusion.

### Predictive Value of Plasma HSP70 to the Diagnosis of AMI, STEMI, and Total Occlusion of Target Vessels

**Figure 3:**
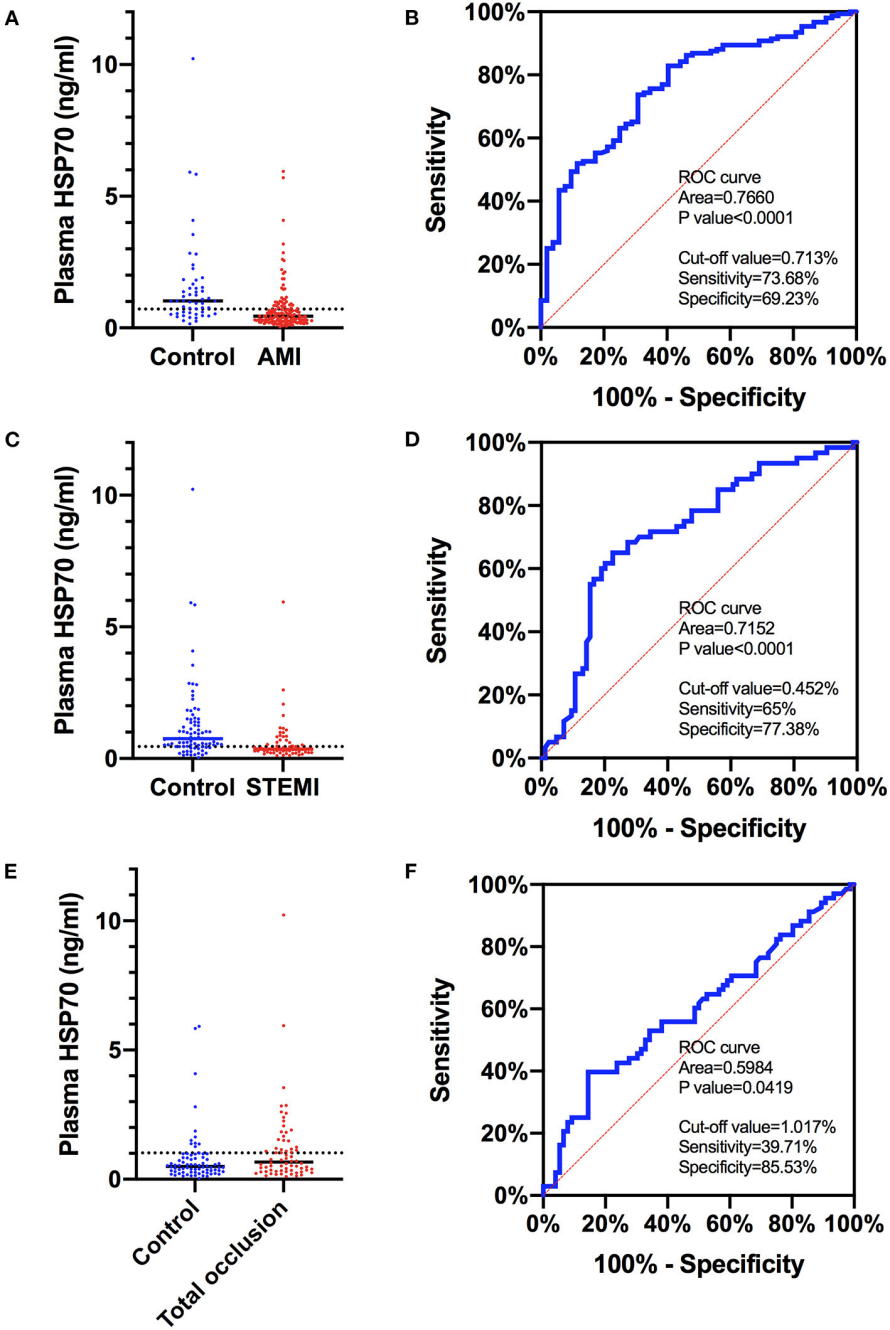
ROC curves of plasma HSP70 to predict AMI, STEMI, and total occlusion in target lesions. **(A)** Comparison of plasma HSP70 in patients with AMI and control patients with no AMI; **(B)** ROC curves of HSP70 to predict AMI, with an AUC of 0.7660 (95% CI 0.6946–0.8374) and best cutoff value of 0.713 ng/ml; **(C)** comparison of plasma HSP70 in patients with STEMI and control patients with no STEMI; **(D)** ROC curves of HSP70 to predict STEMI, with an AUC of 0.7152 (95% CI 0.6289–0.8015) and best cutoff value of 0.452 ng/ml; **(E)** comparison of plasma HSP70 in patients with total occlusion and control patients with no total occlusion; **(F)** ROC curves of HSP70 to predict total occlusion, with an AUC of 0.5984 (95% CI 0.5048–0.6920) and best cutoff value of 1.017 ng/ml. AMI, acute myocardial infarction; STEMI, ST-segment elevation myocardial infarction; ROC, receiver operating characteristic; AUC, area under the curve.

Since HSP70 was negatively correlated with AMI and STEMI, and it was positively correlated with the diagnosis of total occlusion of vessels, the predictive value of plasma HSP70 was evaluated by diagnostic tests (Figure 3). The area under the ROC curve was 0.776 (95% CI 0.6946–0.8374), 0.7152 (95% CI 0.6289–0.8015), and 0.5984 (95% CI 0.5048–0.6920) for prediction of AMI, STEMI, and total occlusion, respectively. The best cutoff value with the largest sum of sensitivity and specificity was 0.713 ng/ml (sensitivity 0.7368, specificity 0.6923), 0.452 ng/ml (0.65, 0.7738), and 1.017 ng/ml (0.3971, 0.8553), correspondingly.

### Plasma HSP70 and In-hospital Outcomes and LVEF at 1-Year Follow-Up

During hospitalization, the incidence of MACE in patients with AMI was 15.2%, which included 14 cases with MACE (Table 5), comprising of 1 patient with non-fatal AMI, 4 with repeat revascularization, 2 with stroke, 4 with malignant ventricular arrhythmia, and 3 with acute left ventricular failure. Besides, there was one patient in the CCS group who confronted with in-hospital non-fatal AMI, which was also added to the total cases with MACE. Logistic regression analysis displayed that plasma HSP70 was not significantly associated with the incidence of in-hospital MACE, whether in patients with only AMI (*P* = 0.4490) or in MI7d along with CCS groups (*P* = 0.4307, Table 6).

**Table 5:** In-hospital outcomes and LVEF at 1-year follow-up in the four groups.

	**MI1d** **(*n* = 60)**	**MI7d** **(*n* = 92)**	**CCS-NCTO** **(*n* = 24)**	**CCS-CTO** **(*n* = 28)**	***P*-value**
In-hospital MACE	3 (5)	14 (15.2)	0 (0)	1 (3.6)	<0.01
In-hospital stay^a^	7 (6, 9)	9 (7, 11)	2 (2, 3)	6 (5, 6.75)	<0.0001
LVEF at 1-year follow-up	54.68 ± 11.24	53.41 ± 10.31	63.75 ± 6.344	55.4 ± 9.709	0.3202

*Values are displayed as mean ± SD or n (%), unless otherwise indicated*.

a *Median and interquartile range*.

*MACE, major adverse cardiovascular events, LVEF, left ventricular ejection fraction*.

**Table 6:** Association analysis of HSP70 and study outcomes in MI7d and CCS groups.

	**OR**	**Spearman *R***	**95% CI**	***P*-value**
In-hospital MACE^a^	0.6624	–	0.2285 to 1.186	0.4307
In-hospital stay	–	−0.2785	−0.4268 to −0.1156	<0.001
In-hospital LVEF	–	0.1615	−0.0076 to 0.3216	0.0540
LVEF at 1-year follow-up	–	0.3281	0.0365 to 0.5683	<0.05

*Correlation of HSP70 and other outcomes were conducted by Spearman correlation analysis*.

*OR, odds ratio; 95% CI, 95% confidence interval; MACE, major adverse cardiovascular events; LVEF, left ventricular ejection fraction*.

a *Simple logistic regression analysis was conducted for HSP70 as potential predictors for MACE*.

As has been reported above, no significant association between HSP70 and in-hospital LVEF was seen (*P* = 0.0540) in MI7d and CCS groups, while HSP70 was positively associated with LVEF at 1-year follow-up (Spearman *R* = 0.3281, 95% CI 0.0365 to 0.5683, *P* < 0.05). On the other hand, the correlation of HSP70 within merely the MI1d group or the MI7d group to LVEF at 1-year follow-up was not significant (*P* = 0.6339 for the MI1d group and *P* = 0.4580 for the MI7d group). Moreover, HSP70 was also negatively correlated with the duration of hospitalization (Spearman *R* = −0.2785, 95% CI −0.4268 to −0.1156, *P* < 0.001). Specifically, as HSP70 was decreased post-MI from day 1 to day 7, the improvement of LVEF at 1-year follow-up was nearly negatively correlated with the reduction of HSP70 during hospitalization (Spearman *R* = −0.4265, 95% CI −0.7516 to 0.0653, *P* = 0.0776), which also indicated that the decreased level of HSP70 during the acute phase of AMI may be involved in improved LVEF at 1 year post-MI.

## Discussion

In this observational study, plasma HSP70 was significantly higher in CCS than in AMI, and HSP70 showed a significant decline during the first week after the onset of AMI, both in patients with STEMI and NSTEMI. Elevated HSP70 was associated with lower diagnosis of AMI and STEMI, decreased levels of LDL-C, peak cTnT, peak NT-proBNP, and peak CRP, but higher diagnosis of total occlusion in target vessels and higher frequency of statin use. HSP70 also showed certain predictive effects on the diagnosis of AMI, STEMI, and total occlusion in lesions, with the area under the ROC curves of the former two above 0.7 and the latter only around 0.6. Regardless of the limited diagnostic ability, there may exist certain pathophysiological mechanisms underlying the correlation between plasma HSP70 and total occlusion.

The role of HSP70 in AMI is controversial. Previous studies have found that the circulating level of HSP70 was elevated upon the onset of AMI and reached the peak value at 4 h and gradually declined in the next few days (5), which is in line with our findings that HSP70 decreased from day 1 to day 7 post-MI. While Dybdahl and colleagues (13) found that HSP70 was higher in patients with AMI than with angina and was positively correlated with cTnT, we found opposite results, which may be explained by our relatively larger sample size and control patients who were more accurately diagnosed with determined coronary lesions by angiography. Previous findings that HSP70 may mediate proinflammatory responses during ischemic injury (14) were also contrary to our results, which may be attributed to the different ways to evaluate ischemic inflammation. In detail, Satoh et al. estimated local inflammation via *in vitro* studies to examine the phenotype of HSP70-stimulated monocytes (14), while we chose a marker of systemic inflammation, CRP. Therefore, it seemed that HSP70 does not necessarily act as DAMPs to provoke strong inflammation and expand the ischemic damage after AMI, given its negative association with markers indicating severity of myocardial injury as well as in-hospital stay and positive correlation with LVEF at 1-year follow-up, despite no correlation with in-hospital MACE. However, Bochaton et al. found that HSP70 was associated with adverse cardiac events and a worse prognosis of MI at 18 months, and it may implicate that HSP70 is detrimental in the long run. Thus, whether HSP70 might be a stabilizer during the early phase of MI or a disturber in the long-term prognosis deserves further investigation. Although the diagnostic ability of HSP70 in AMI and STEMI was moderate (area under the curve was 0.77 and 0.72, respectively), the circulating levels of HSP70 may be a marker that assists in the diagnosis of acute chest pain.

The protective roles of circulating HSP70 in AMI have been supported by various molecular and *in vivo* studies. It is noted that these functional studies of HSP70 mainly focus on its intracellular level rather than extracellular concentration, which may lead to divergent conclusions. Anyway, the downstream mechanisms mediating the effects of intracellular or extracellular levels of HSP70 are not entirely distinct. HSP70 is supposed to act as molecular chaperons to retard protein conformational abnormalities implicated in atherosclerosis as well as myocardial IR, (22) and it is also considered as DAMP to interact with classic pattern recognition receptors (PRRs) such as TLR2 and TLR4 (3). As has been debated, it remains uncertain whether HSP70 is virtually involved in the inflammation after MI in humans or is a bystander which has no causal relationship with MI (3), and future studies are warranted.

The dynamic change in the level of HSP70 at different time points of AMI is displayed in our findings, as HSP70 decreased from the onset of AMI during a week and might gradually increase to a certain level when the AMI lesion developed into CTO lesion. The decrease of HSP70 from day 1 to day 7 after the acute insult of MI may result from circulating HSP70 shifting into the dying cells at the ischemic site, imposing intracellular protective effects, which remains to be validated. The formation of CTO lesion is with concomitant coronary collateral circulation development (23), which to some extent offers benefits for sustaining the oxygen supply in ischemic sites of the myocardium. Besides, the complex mechanisms underlying CTO formation also underscore the long-term ischemia-related myocardial stunning (23), which is characterized by the altered expression of metabolic and prosurvival proteins (24). Thus, the increased level of HSP70 from the acute phase of infarction to CTO may implicate the possible “prosurvival” role of HSP70 in the development of collateral connections and myocardial hibernation, which needs to be proven in experimental models.

Notably, the association of plasma HSP70 and total occlusion in target vessels has not been reported previously. CTO is one of the most complex and difficult coronary lesion types that interventional cardiologists have to deal with, which is commonly formed over months after acute occlusion of coronary vessels. Different from acute occlusion lesions, it seemed that collateral circulation is more developed and the overall hemodynamics of patients is more stable in CTO. We discovered that HSP70 was positively correlated with and restrictively predicted the diagnosis of total occlusion (area under the curve 0.60), which implicates that HSP70 may be involved in the development of total occlusion. It has been reported that HSP70 levels correlate with lesion severity in apolipoprotein E-null mice (25). In addition, HSP70 may promote arterial calcification and mineralization via acting as a matrix Gla protein (MGP)-binding protein, which is the inhibitor of vascular calcification (4). These findings suggest the possible role of HSP70 in the active formation of solid and stable lesions rather than soft and vulnerable plaques during atherosclerosis.

## Study Limitations

First, our study population was limited to patients with AMI and CCS, while patients with unstable angina were not included. Second, patients with CCS were enrolled consecutively and respectively in CCS-NCTO and CCS-CTO in order to guarantee adequate samples in the CTO group, and were not matched with those from AMI groups, which may cause certain selective bias in statistical analysis. It seemed that patients in the CCS-CTO group were especially younger and had a higher frequency of previous MI as well as lower HDL-C levels, which may be attributed to the pathophysiology of CTO lesions. Third, the sample size of the study was relatively small; thus, the results need careful interpretation. Fourth, the diagnostic values of HSP70 evaluated by the ROC curve were moderate and restricted and could only assist the diagnosis. Finally, the markers selected to assess the severity of myocardial infarction, inflammation, and cardiac function were not specific and virtually accurate, which calls for more delicate approaches to evaluate the association of HSP70 and severity of AMI.

## Conclusions

In this observational study concerning patients with acute myocardial infarction and chronic coronary syndrome with or without total occlusion of target lesions, plasma circulating levels of HSP70 were found to be negatively associated with the severity of myocardial ischemic injury and in-hospital stay and positively correlated with LVEF at 1-year follow-up for patients with acute symptoms. To some extent, these findings clarified the roles of circulating HSP70 in AMI. Besides, the ability of HSP70 to identify clinically significant AMI and STEMI is moderate, and the predictive value to total occlusions is slight.

## Data Availability Statement

The datasets presented in this article are not readily available. Requests to access the datasets should be directed to the Ministry of Science and Technology of the People's Republic of China, http://www.most.gov.cn/eng/.

## Ethics Statement

The study's involving human participants were reviewed and approved by Ethics Committee of Zhongshan Hospital, Fudan University. The patients/participants provided their written informed consent to participate in this study.

## Author Contributions

KY contributed to the conceptualization, methodology, and review of writing and editing. JG contributed to the conceptualization, methodology, and supervision. RW contributed to the conceptualization, data collection, investigation, and writing of the original draft. WG contributed to the methodology, software, investigation, and data curation. ZD contributed to the data collection and investigation. YS contributed to the investigation and data curation. YJ contributed to the investigation and data curation. JL contributed to the formal analysis. YM contributed to the data collection and curation. YD contributed to the methodology and supervision. KY and JG are responsible for the overall content as guarantors.

## Funding

This study was supported by the National Key Research and Development Program of China (2016YFC1301202) from the Ministry of Science and Technology of the People's Republic of China.

## Conflict of Interest

The authors declare that the research was conducted in the absence of any commercial or financial relationships that could be construed as a potential conflict of interest.

## Publisher's Note

All claims expressed in this article are solely those of the authors and do not necessarily represent those of their affiliated organizations, or those of the publisher, the editors and the reviewers. Any product that may be evaluated in this article, or claim that may be made by its manufacturer, is not guaranteed or endorsed by the publisher.
